# Supplementary material for: Single-strand DNA processing: phylogenomics and sequence diversity of a superfamily of potential prokaryotic HuH endonucleases
Source: BMC Genomics. 2018 Jun 19;19:475. doi: 10.1186/s12864-018-4836-1 (PMC6006769; doi:10.1186/s12864-018-4836-1)
Supplement: Supplementary file 1 — Figure S1. Distribution of protein length in each subclass; Figure S2. Coverage of HMM profiles and proteins for each subclass; Figure S3. ab initio search for conserved motifs with MEME in proteins of each subclass; Figure S4. Distribution of the distances (AA) between the HuH and Y motifs in proteins of each subclass; Figure S5. Genome size and the occurrence of TnpAY1; Figure S6. Short repeated sequences in subclass 2.4 TnpAREP; Figure S7. Homology modelling of proteins with extra domains in the conserved core domain; Figure S8. C-terminal subclass specific domains; Figure S9. Additional subclass specific domains; Figure S10. Subclass 2.4, REP insertions in coding sequences; Figure S11. Analysis of conservation in subclasses of the key residues involved in 5′ GTAG guide sequence binding; Figure S12. Number of intra genomic copies of each subclass; Figure S13. Percentage of sequence alignment identities between pairs of TnpAY1 sequences. (DOCX 2899 kb) [file 12864_2018_4836_MOESM1_ESM.docx]

**Supplementary figures**

**
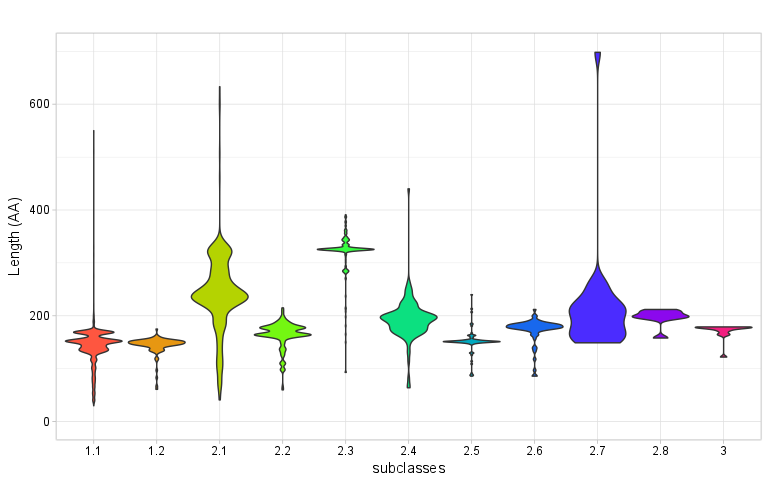
**

**Figure S1: Distribution of protein length in each subclass.** The violin plots contain all of the observed data and provide important information about the distribution of data. Along the length axis, the width of the violin is proportional to the density of proteins sharing the same size.


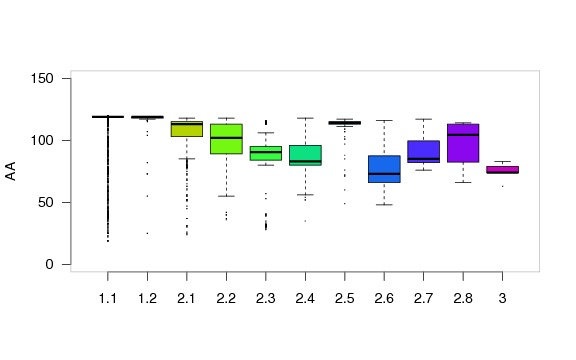

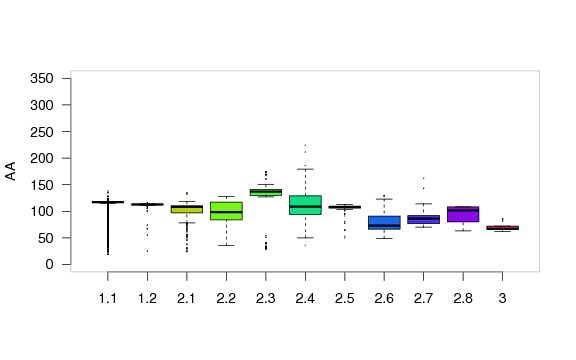


A

B


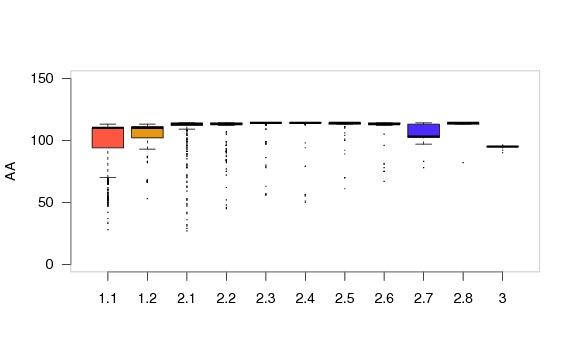

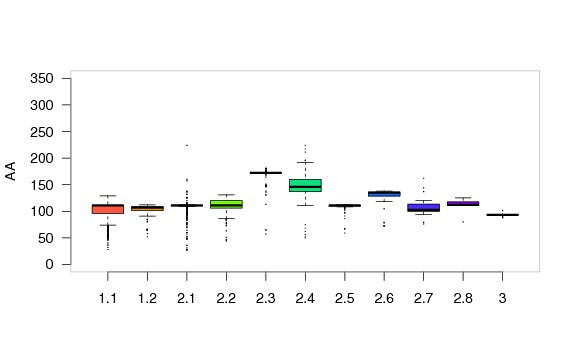


C

D

**Figure S2: Coverage of HMM profiles and proteins for each subclass.** Panels A and B, alignment of Y1_Tnp Pfam profile (PF01797) with TnpA_Y1_. Length distribution of the Y1_Tnp profile (A) and of the proteins (B) in the alignment. Panels C and D, alignment of TnpA_REP_-like profile with TnpA_Y1_. Length distribution of the TnpA_REP_-like profile (C) and of the proteins (D) in the alignment.

**Figure S3: *ab initio* search for conserved motifs with MEME in proteins of each subclass.** Subclass 2.8 was too small for relevant motif discovery so it was enlarged with blastp hits found in the NCBI nr database. In addition to HuH, W and Y motifs, those identified in protein N- or C-term are reported. Subsequences with the MEME motifs were extracted with few flanking residues and aligned on the inter-subclasses conserved residues. Amino acid conservation is presented as sequence logos.


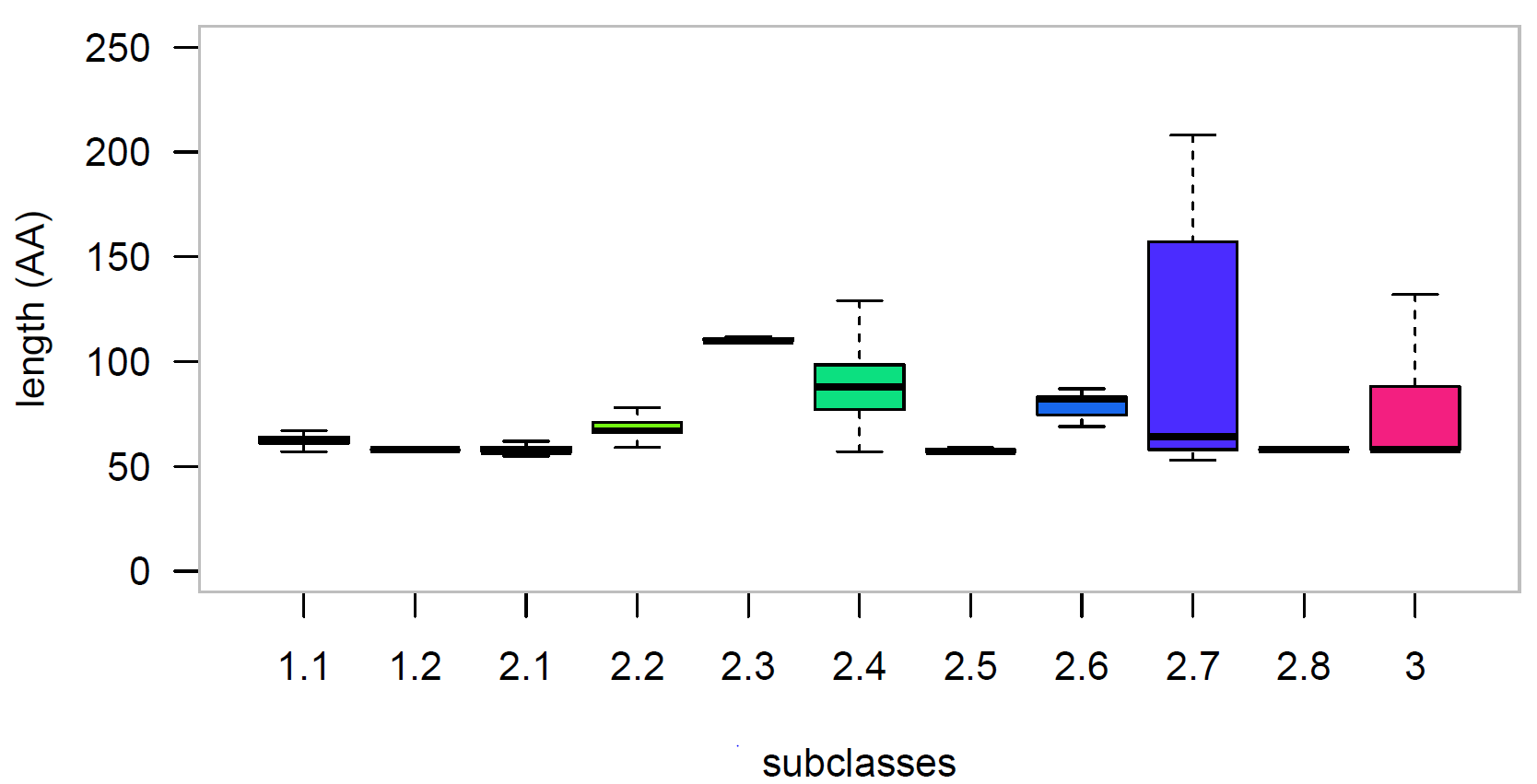


**Figure S4: Distribution of the distances (AA) between the HuH and Y motifs in proteins of each subclass.**

**
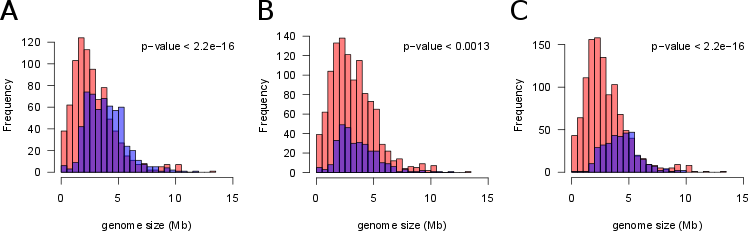
Figure S5: Genome size and the occurrence of TnpA_Y1_.** A, distribution of the genome size for genomes that encode at least one subclass member (blue) or none (orange). B, same as A but only subclass 1.1 is considered and C all except subclass 1.1 are considered.

**Figure S6: Short repeated sequences in subclass 2.4 TnpA_REP_.** The repeats are predicted with MEME. Protein sequences are displayed as grey rectangles, the green boxes referred to the N-term and C-term regions of the conserved core domain. The repeats are shown as red and blue boxes.


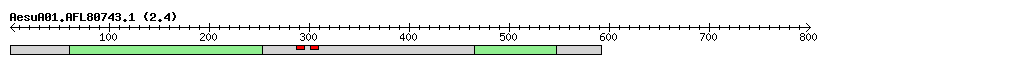

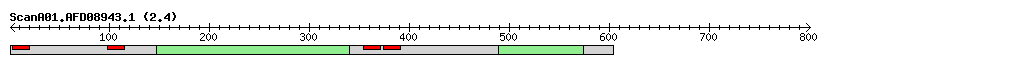

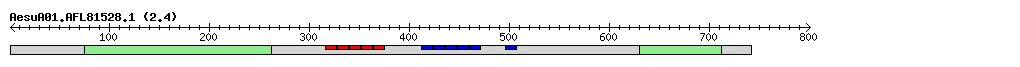

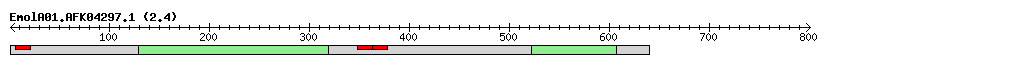

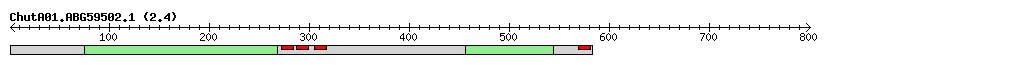

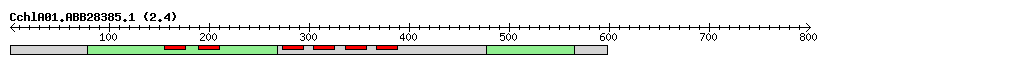

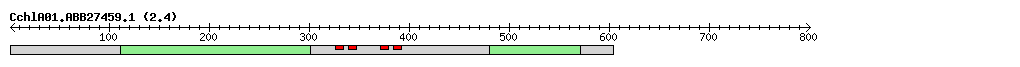

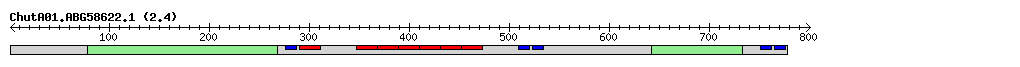

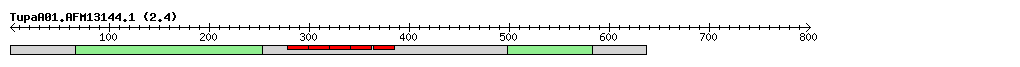

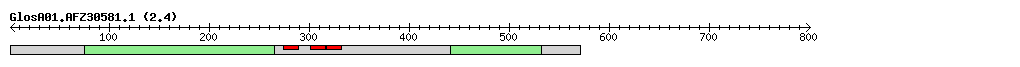

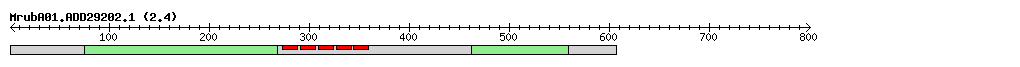

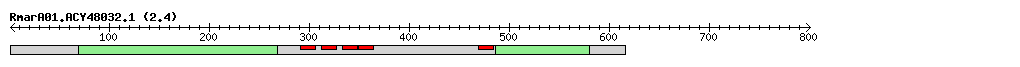

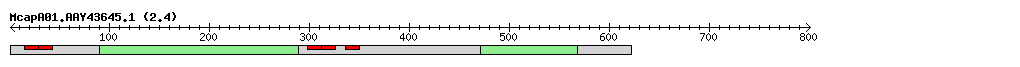

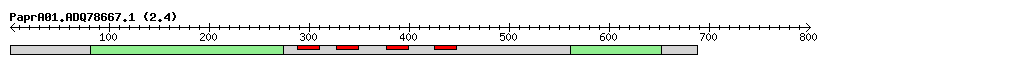

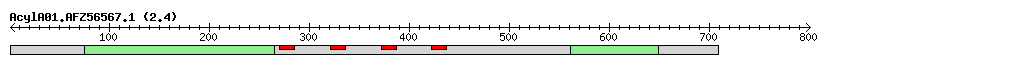

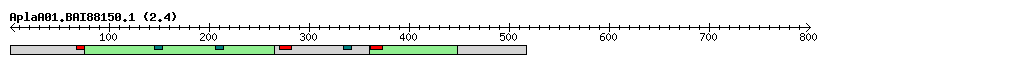

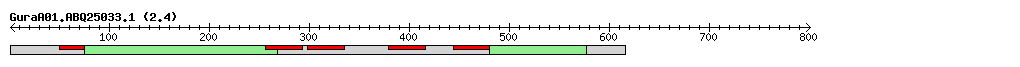

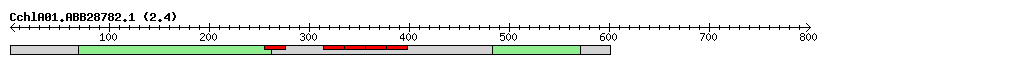

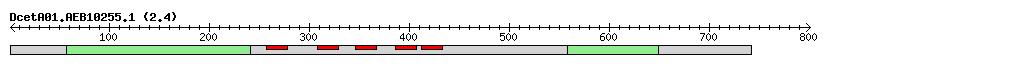

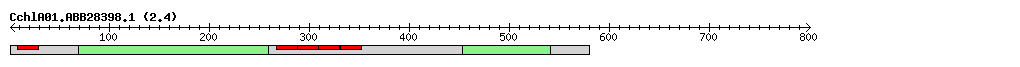

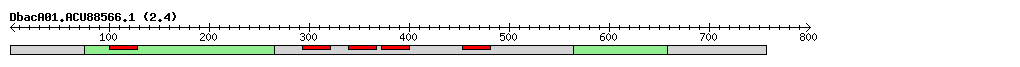

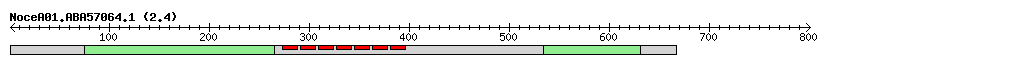

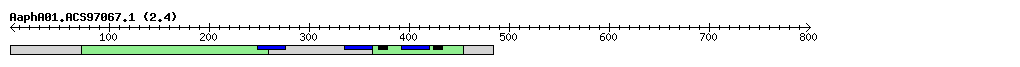

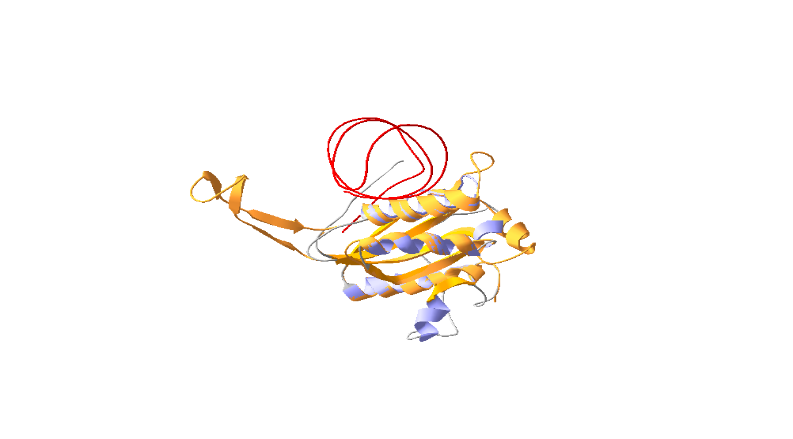

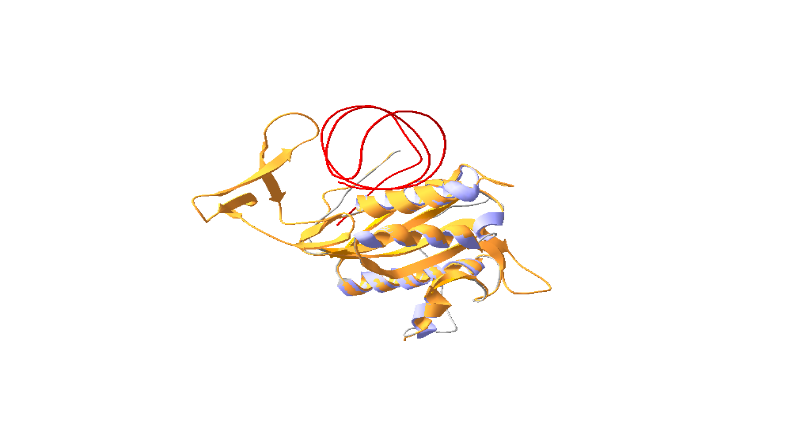


subclass 2.3

subclass 2.4

β3/αC loop

β3/αC loop

β1/αA loop

αB loop

A

B


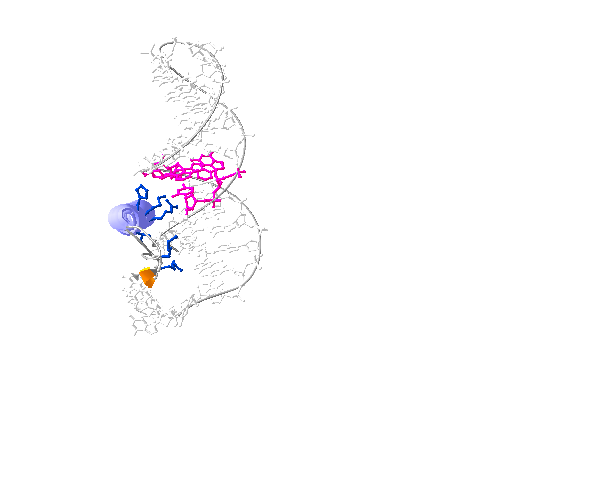


canonical

C

Arg 97

Lys 91

His 86

Thr 85

Lys 82

Arg 98

A 12

A 13

G 26

C 27


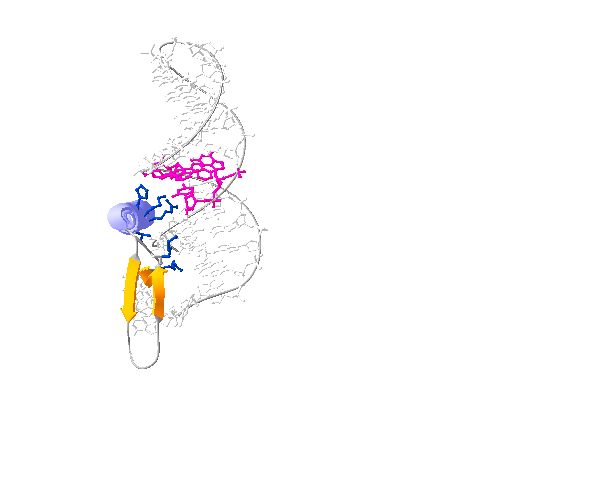


subclass 2.2

D

extra domain


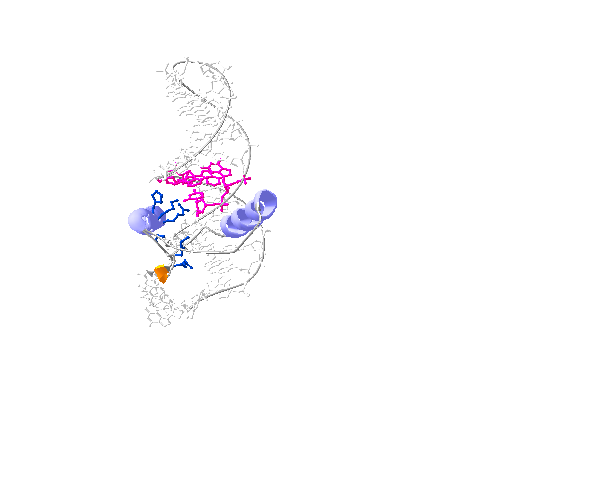


subclass 2.6

E

extra domain

**Figure S7: Homology modelling of proteins with extra domains in the conserved core domain.** The structure of REP bound to *E. coli* TnpA_REP_ (PDB: 4ER8) is used as a model (blue). The predicted structure (yellow) is superimposed onto the crystal structure of 4ER8 (blue), REP sequence in red. Panel A, subclass 2.3 protein ACR12284 (C5BUM5) from *Teredinibacter turnerae* T7901 and Panel B subclass 2.4 protein AFY32102 (K9PHU2) from *Calothrix sp.* PCC 7507. The extra domains (blue arrows) are inserted in a loop between β3 and αC. Panel C, a zoom of the 4ER8 protein region involved in interactions with the REP sequence. Color code: alpha helix in blue, beta sheet in yellow, REP in light grey and nucleotides present in REP bulge in magenta. Panel D, subclass 2.2 protein ADE15902 (D5BXZ1) from *Nitrosococcus halophilus* Nc 4, the extra domain is predicted as antiparallel β sheets and E, subclass protein 2.6 AEA44309 (F2IBK1) from *Fluviicola taffensis* DSM 16823, the extra domain is predicted as an α helix. Swiss PDB viewer was used for structure analysis. The homology modeling was accomplished using SWISS-MODEL and the visualization with Swiss-PdbViewer (Guex *et al.* 2009).


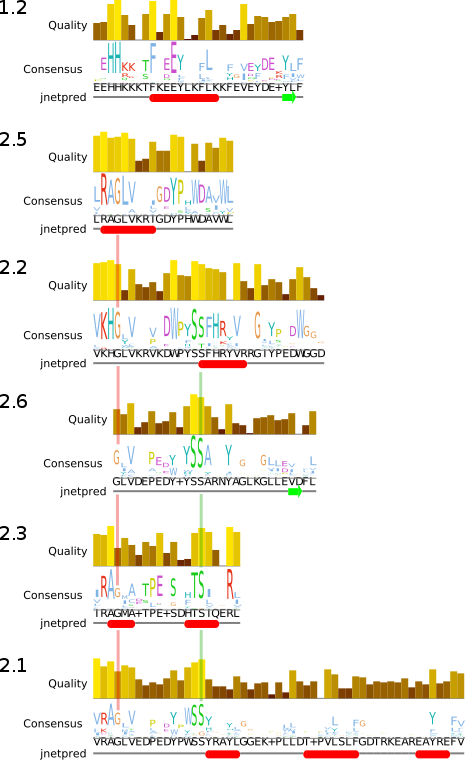


**Figure S8: C-terminal subclass specific domains.** Jalview quality and consensus annotations and secondary predictions (Jnetpred server) are reported for each domain (red, alpha helix and green beta sheet). Inter domain conserved residues are link with orange and green lines.

B

A

**Figure S9: Additional subclass specific domains.** See legend Figure S8.


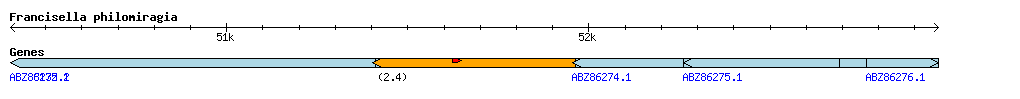

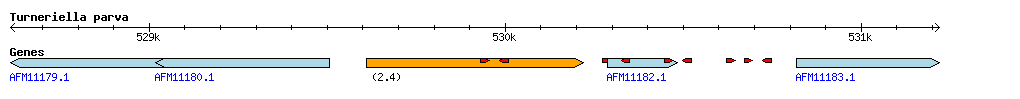

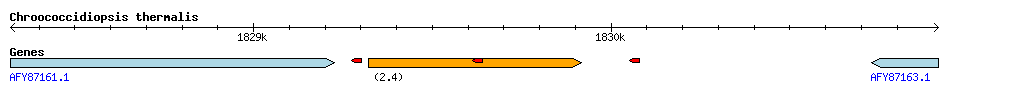

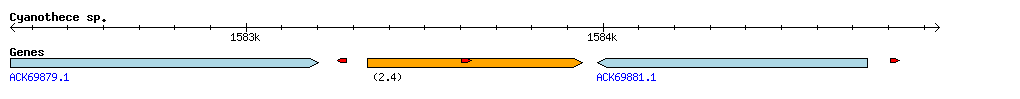

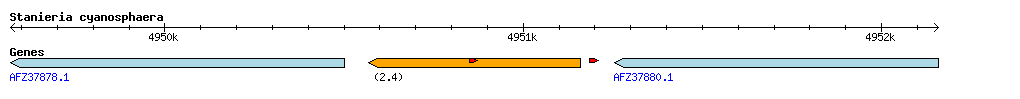

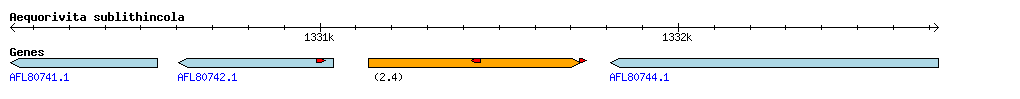

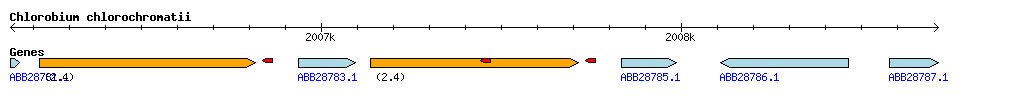

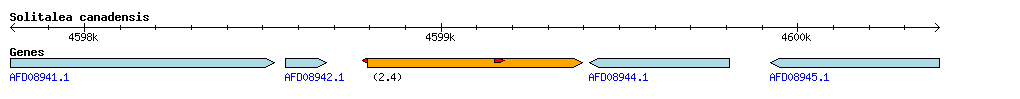

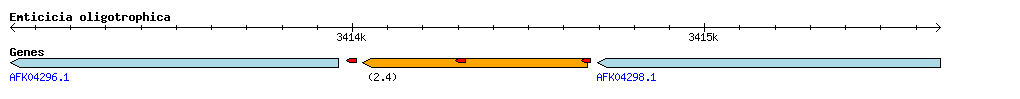

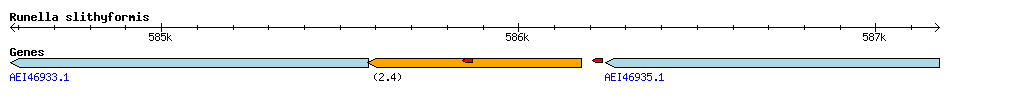

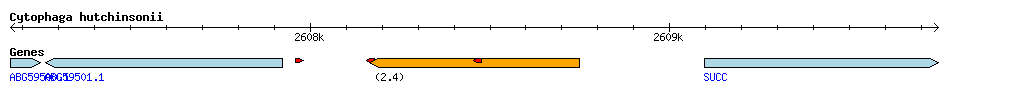

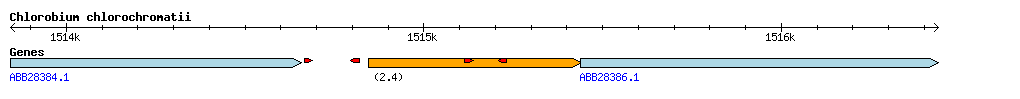

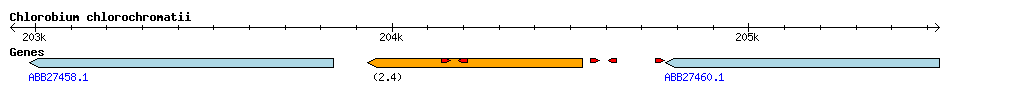

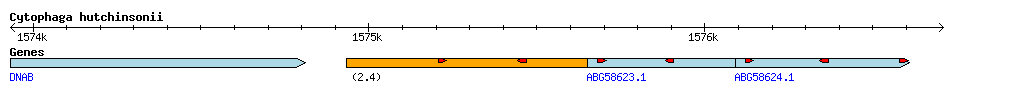

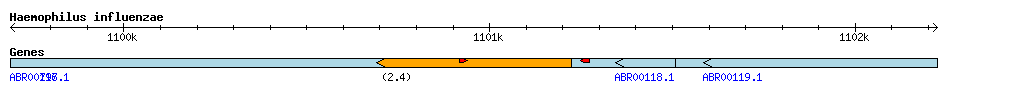

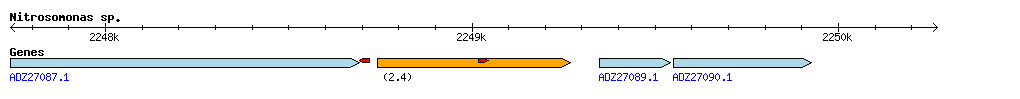

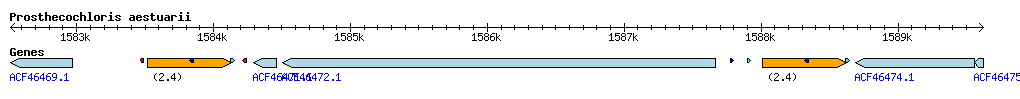

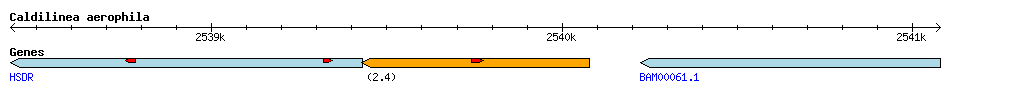

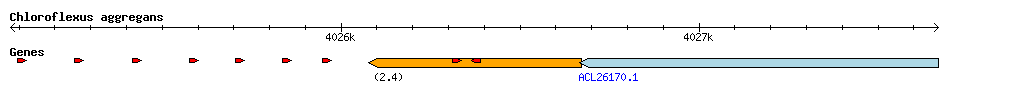

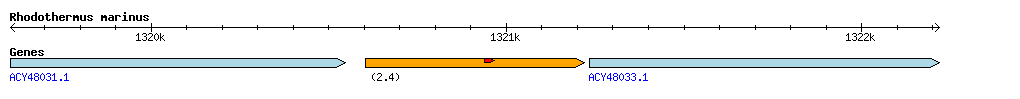

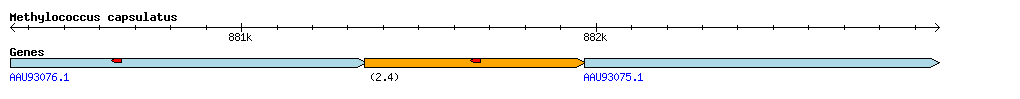

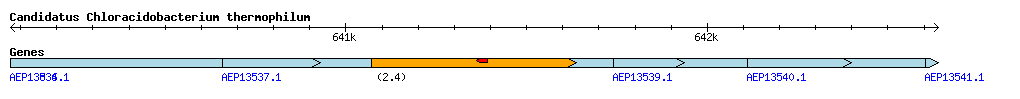

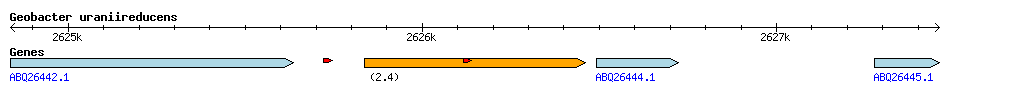

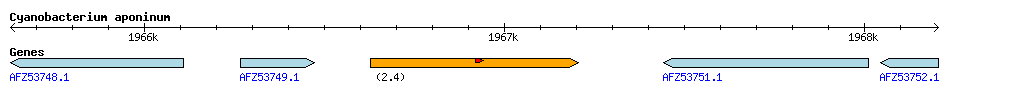

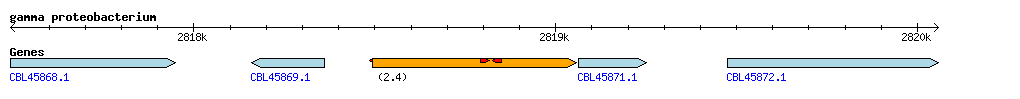

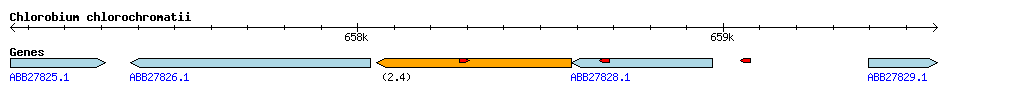

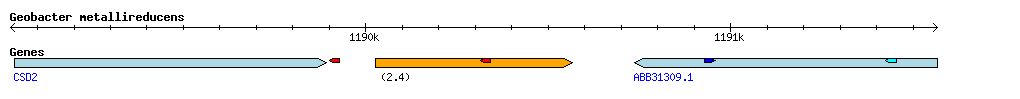

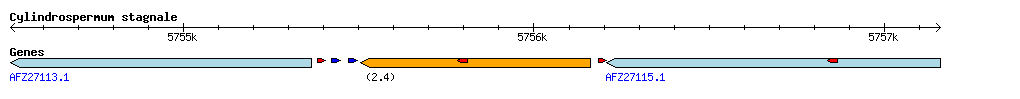

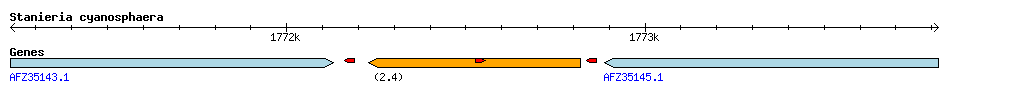


**Figure S10: Subclass 2.4, REP insertions in coding sequences.** The *tnpA*_REP_ gene (orange) is flanked by genes (blue). REP sequences are annotated as small red colored arrows.


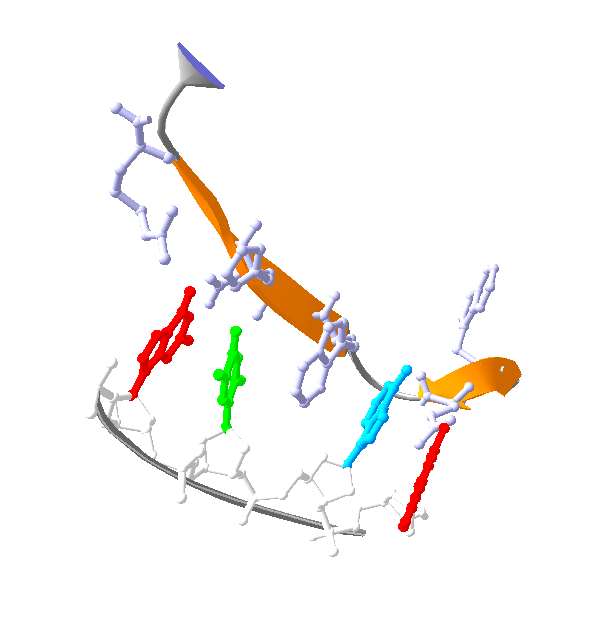


G 1

T 2

A 3

G 4

R104

H101

W99

Q95

E100

W94


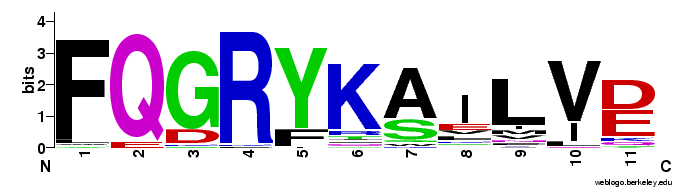


2.1.2


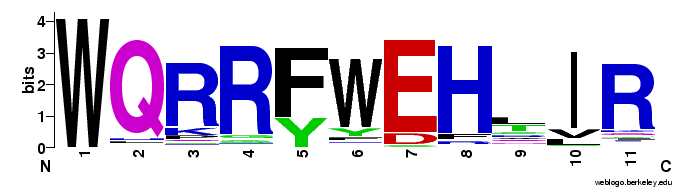


2.2


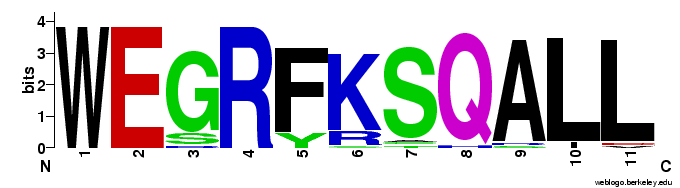


2.3


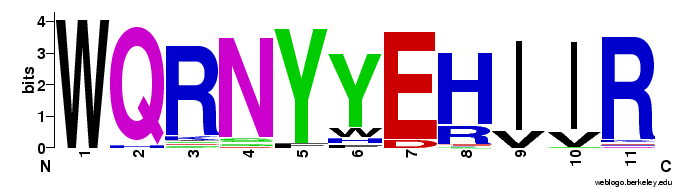


2.4


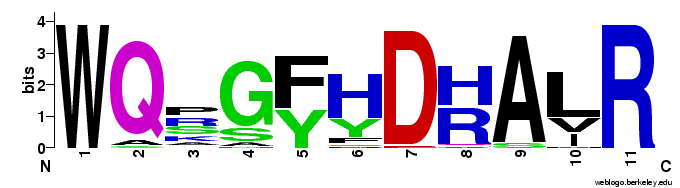


2.5


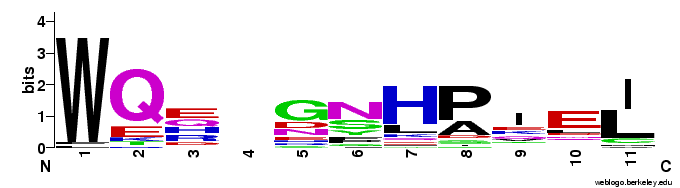


2.6


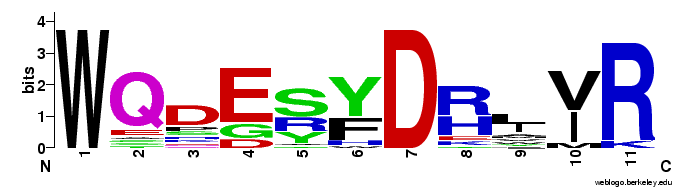


2.7


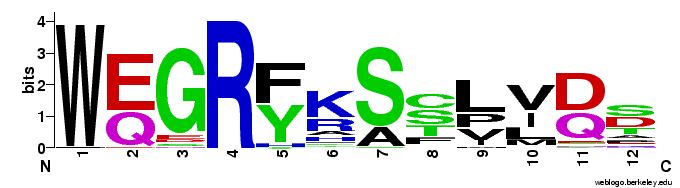


2.1.1

A

B

94

95

99

100

101

104

**Figure S11: Analysis of conservation in subclasses of the key residues involved in 5’ GTAG guide sequence binding.** A, interactions reported for *E. coli* TnpA_REP_. B, sequence logos of the homologous region in each subclass. Position refers to *E. coli* TnpA_REP_ coordinates


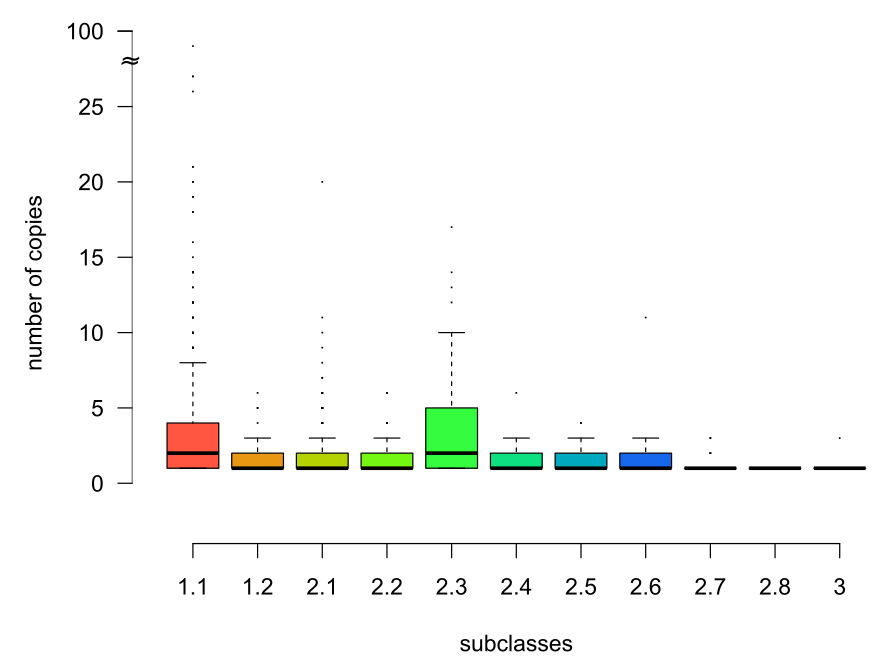


**Figure S12: Number of intra genomic copies of each subclass.**

**
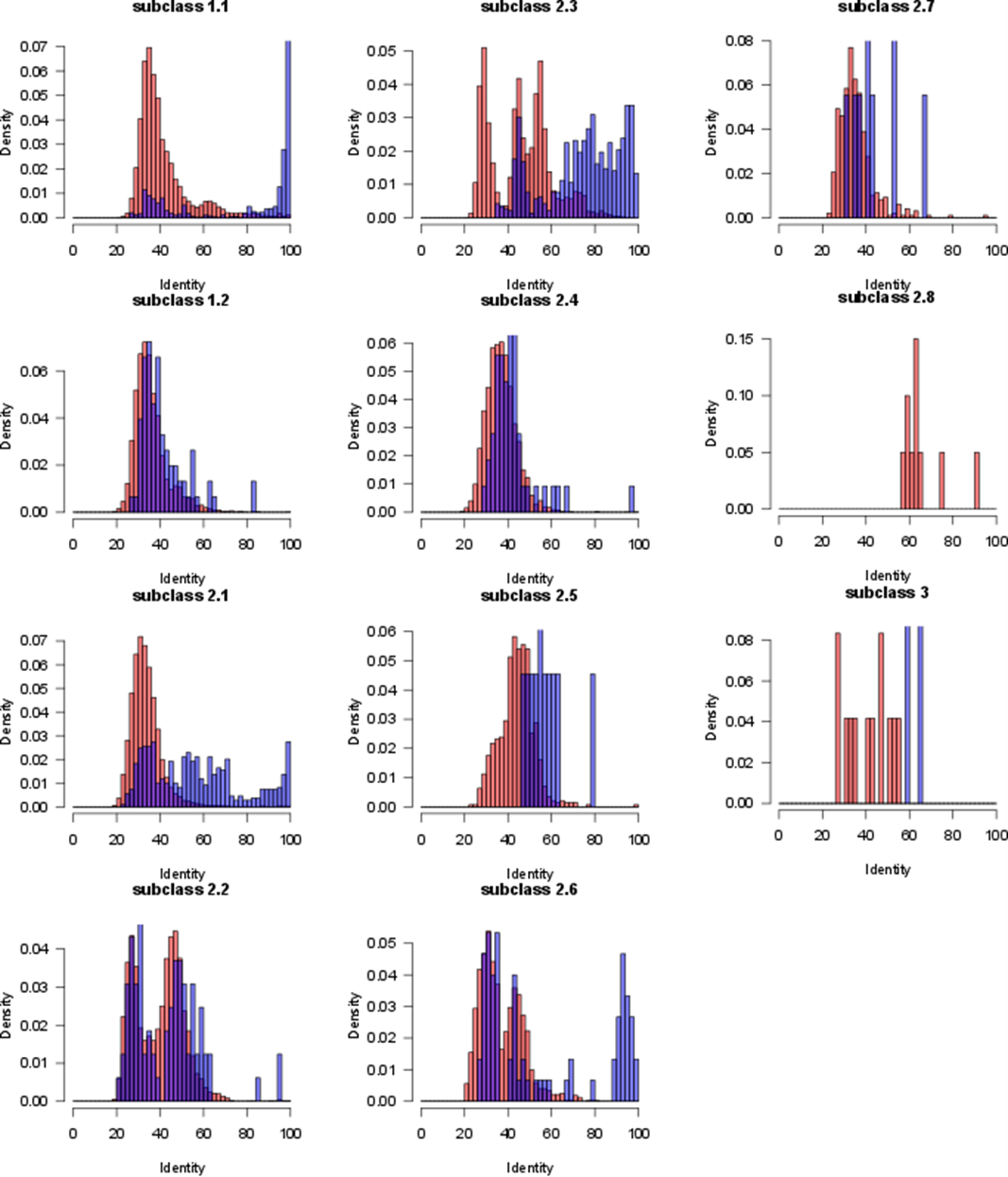
**

**Figure S13: Percentage of sequence alignment identities between pairs of TnpA_Y1_ sequences.** A plot is shown for each subclass: blue, intra-genomic copies, orange, inter-genomic copy comparisons.
